# 1266. Effects of Rapid Initiation of Antiretroviral Therapy in an Urban Clinic Setting

**DOI:** 10.1093/ofid/ofac492.1097

**Published:** 2022-12-15

**Authors:** Daniel Mesa, Yae Ji Kim

**Affiliations:** University of Maryland School of Pharmacy, Sayreville, New Jersey; SUNY Downstate Medical Center, New York, New York

## Abstract

**Background:**

Rapid initiation of antiretroviral therapy (ART), i.e. within 72 hours of diagnosis, in people living with HIV (PLWH) has shown improved viral suppression, medication adherence, and retention in care, compared to deferral until initial labs have resulted. More real-world experience with rapid ART programs is needed, however, particularly among clinics in resource-limited settings that serve a complex and largely uninsured or underinsured population, such as the HIV clinic at SUNY Downstate Medical Center. In 2017, the clinic implemented rapid ART in select PLWH.

**Methods:**

This was a single-center retrospective chart review of patients seen at our HIV clinic between January 2015 and June 2021. Patients were included if they were ART-naïve or off ART for > 3 months. Exclusion criteria included baseline undetectable viral load (VL) and perinatal HIV infection. Patients were assigned to standard or rapid start groups based on time from intake to initiation of ART. The primary outcome was proportion of patients with VL < 50 copies/mL at week 52. Secondary outcomes were proportion of patients with VL < 200 copies/mL, retention in care, and mean weight change from baseline, all at week 52; time from intake to ART; and time to viral suppression.

**Results:**

As shown in Table 1, of the 190 patients that were included, 133 were rapid start while 77 were standard start. 103 patients had baseline resistance data; only 1 had integrase stand inhibitor resistance. Median time to ART was 0 days and 2.5 weeks in the rapid and standard groups, respectively. The most common initial regimen was BIC/FTC/TAF in both groups (49.6%, 31.2%). As described in Tables 2 and 3, 62.1% and 73.7% of patients had VL < 50 and < 200 copies/mL at week 52, respectively. Nearly 73% of patients remained in care at week 52, with similar rates between groups. Time from ART to viral suppression was similar between groups.

**Figure:**
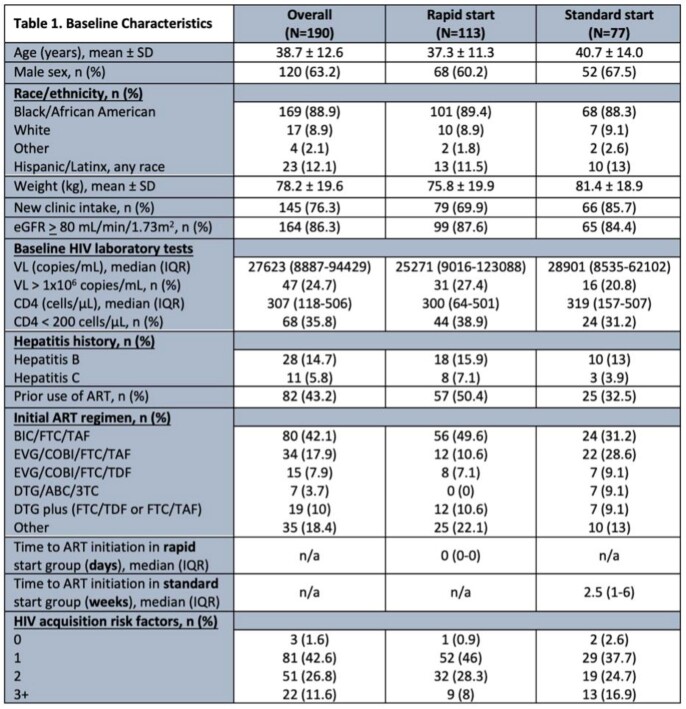

**Figure:**
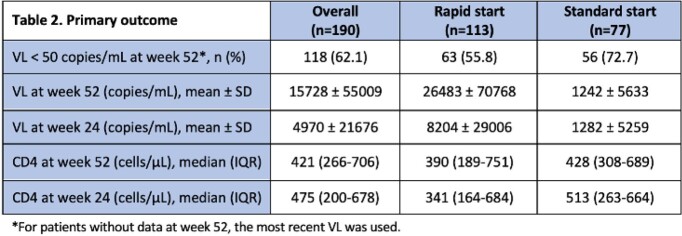

**Figure:**
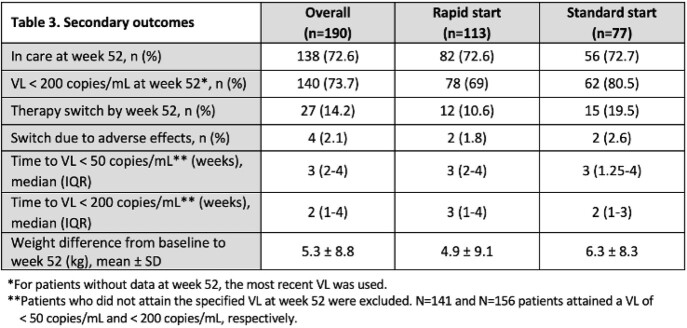

**Conclusion:**

Rapid ART was well-tolerated but resulted in similar viral suppression and retention in care at week 52 compared to standard ART. However, rapid ART resulted in quicker viral suppression, although further evaluation of the clinical impact of this finding is needed. This study highlights the need for a multifaceted approach to engaging patients with HIV throughout the care continuum to ensure retention in care.

**Disclosures:**

**All Authors**: No reported disclosures.

